# A quantitative systems pharmacology model for certolizumab pegol treatment in moderate-to-severe psoriasis

**DOI:** 10.3389/fimmu.2023.1212981

**Published:** 2023-09-20

**Authors:** Pablo Coto-Segura, Cristina Segú-Vergés, Antonio Martorell, David Moreno-Ramírez, Guillem Jorba, Valentin Junet, Filippo Guerri, Xavier Daura, Baldomero Oliva, Carlos Cara, Olaya Suárez-Magdalena, Sonya Abraham, José Manuel Mas

**Affiliations:** ^1^ Dermatology Department, Hospital Vital Alvarez-Buylla de Mieres, Asturias, Spain; ^2^ Anaxomics Biotech SL, Barcelona, Spain; ^3^ Structural Bioinformatics Group, Research Programme on Biomedical Informatics, Department of Medicine and Life Sciences, Universitat Pompeu Fabra, Barcelona, Spain; ^4^ Dermatology Department, Hospital de Manises, Valencia, Spain; ^5^ Dermatology Department, University Hospital Virgen Macarena, Andalusian Health Service, University of Seville, Seville, Spain; ^6^ Institute of Biotechnology and Biomedicine, Universitat Autònoma de Barcelona, Cerdanyola del Vallès, Spain; ^7^ Catalan Institution for Research and Advanced Studies (ICREA), Barcelona, Spain; ^8^ Centro de Investigación Biomédica en Red de Bioingeniería, Biomateriales y Nanomedicina, Instituto de Salud Carlos III, Cerdanyola del Vallès, Spain; ^9^ Medical Affairs, UCB Pharma, Madrid, Spain; ^10^ National Heart and Lung Institute (NHLI), Faculty of Medicine, Imperial College, London, United Kingdom; ^11^ Medical Affairs, UCB Pharma, Brussels, Belgium

**Keywords:** psoriasis, anti-TNF, certolizumab pegol, mathematical modelling, virtual population, mechanism of action

## Abstract

**Background:**

Psoriasis is a chronic immune-mediated inflammatory systemic disease with skin manifestations characterized by erythematous, scaly, itchy and/or painful plaques resulting from hyperproliferation of keratinocytes. Certolizumab pegol [CZP], a PEGylated antigen binding fragment of a humanized monoclonal antibody against TNF-alpha, is approved for the treatment of moderate-to-severe plaque psoriasis. Patients with psoriasis present clinical and molecular variability, affecting response to treatment. Herein, we utilized an *in silico* approach to model the effects of CZP in a virtual population (vPop) with moderate-to-severe psoriasis. Our proof-of-concept study aims to assess the performance of our model in generating a vPop and defining CZP response variability based on patient profiles.

**Methods:**

We built a quantitative systems pharmacology (QSP) model of a clinical trial-like vPop with moderate-to-severe psoriasis treated with two dosing schemes of CZP (200 mg and 400 mg, both every two weeks for 16 weeks, starting with a loading dose of CZP 400 mg at weeks 0, 2, and 4). We applied different modelling approaches: (i) an algorithm to generate vPop according to reference population values and comorbidity frequencies in real-world populations; (ii) physiologically based pharmacokinetic (PBPK) models of CZP dosing schemes in each virtual patient; and (iii) systems biology-based models of the mechanism of action (MoA) of the drug.

**Results:**

The combination of our different modelling approaches yielded a vPop distribution and a PBPK model that aligned with existing literature. Our systems biology and QSP models reproduced known biological and clinical activity, presenting outcomes correlating with clinical efficacy measures. We identified distinct clusters of virtual patients based on their psoriasis-related protein predicted activity when treated with CZP, which could help unravel differences in drug efficacy in diverse subpopulations. Moreover, our models revealed clusters of MoA solutions irrespective of the dosing regimen employed.

**Conclusion:**

Our study provided patient specific QSP models that reproduced clinical and molecular efficacy features, supporting the use of computational methods as modelling strategy to explore drug response variability. This might shed light on the differences in drug efficacy in diverse subpopulations, especially useful in complex diseases such as psoriasis, through the generation of mechanistically based hypotheses.

## 1 Introduction

Psoriasis is a chronic immune-mediated inflammatory systemic disease with skin manifestations typically characterized by erythematous, scaly, itchy and/or painful plaques. The physiopathology of psoriasis is marked by a complex molecular interplay involving dysregulated cytokines, immune cell activation, and altered keratinocyte proliferation, contributing to the development and persistence of the disease (1, 2). Psoriasis affects 1-4% of the population worldwide (3–5). Patients with psoriasis generally present a significantly reduced health-related quality of life and a high burden of disease (6–10). Psoriasis is often linked to comorbidities, especially in its moderate-to-severe forms. These can include psoriatic arthritis, cardiometabolic diseases, metabolic syndrome, obesity, and depression (5, 11–13), highlighting the systemic nature of the disease and the importance of multidisciplinary care. The molecular mechanisms underlying the relationship between psoriasis and its comorbidities are not fully elucidated, underscoring the need for additional research to unravel the interplay between these conditions at a molecular level (14). This could affect treatment efficacy, urging the need to address psoriasis’ treatment in personalized clinical setting (15, 16). Current treatment for moderate-to-severe psoriasis includes phototherapy, oral systemic immunomodulatory drugs (methotrexate, apremilast, acitretin, and cyclosporine), and biologic agents (17, 18). The latter are monoclonal antibodies often administered subcutaneously that inhibit different cytokines, including tumor necrosis factor (TNF), interleukin (IL)-12/23, IL-17s, and IL-23.

Certolizumab pegol [CZP] is a PEGylated antigen binding fragment (Fab’) of a humanized monoclonal antibody against TNF-alpha. CZP is currently approved for the treatment of moderate-to-severe psoriasis, psoriatic arthritis, axial spondylarthritis, rheumatoid arthritis, and Crohn’s disease (Crohn’s disease is only approved by the FDA) (19–21). CZP has shown a rapid and sustained reduction of psoriasis activity and improvement in patients’ quality of life in pivotal studies CIMPASI-1, CIMPASI-2 and CIMPACT (22–25), with a favorable safety profile (26–28). Due to its molecular structure without constant fragment (Fc-free), CZP has no to minimal transfer from mother to infant across the placenta and to breast milk, and is the only biologic agent with pharmacokinetic clinical data in its label supporting potential use in both pregnancy and breastfeeding for chronic inflammatory diseases (29–31). The conjugation of the Fab’ fragment to two molecules of polyethylene glycol (PEG) has been associated to increased half-life and reduced antigenicity, immunogenicity, and toxicity (32). It has also been linked to enhanced selectivity for inflamed tissue compared to non-inflamed tissue. CZP has shown more rapid tissue penetration, higher levels, and greater persistence in inflamed tissue when compared to adalimumab and infliximab (33, 34).

In Europe, the approved CZP dosing schedule for the treatment of plaque psoriasis consists of a loading dose of CZP 400 mg (given as 2 subcutaneous injections of 200 mg each) at Weeks 0, 2 and 4, and a maintenance dose of CZP 200 mg Q2W. In patients with insufficient response, a dose of 400 mg Q2W can be considered (21). In the United States, the FDA approved CZP dosing schedule consists of 400 mg Q2W (given as 2 subcutaneous injections of 200 mg each). For some patients (with body weight ≤90 kg), a dose of 400 mg initially and at Weeks 2 and 4, followed by 200 mg every other week may be considered (20). From analysis of the baseline characteristics of patients included in CIMPASI-1, CIMPASI-2 and CIMPACT, it is not possible to identify which patients would most benefit from the CZP 400 mg Q2W dose. However, for almost all measures, CZP 400 mg Q2W demonstrates numerically superior efficacy results compared to CZP 200 mg Q2W. Interpatient variability in treatment response poses challenges in managing psoriasis. Molecular heterogenicity significantly contributes to this variability, highlighting the necessity of a better understanding of the molecular factors that underlie such differences (35, 36).

Modelling and computational techniques are increasingly being used in biomedical investigation. These approaches offer valuable tools for investigating complex issues that are challenging to test in live organisms, as well as for generating mechanistic hypotheses to elucidate clinical observations. The insights gained through modeling can then be further validated and contrasted with *in vitro* or *in vivo* experimental results, enhancing our understanding of biological processes, and facilitating advancements in medical science. They can also address methodological challenges, bridging the gap between randomized clinical trials (RCTs) and observational studies (37, 38). In fact, the US and European medicines agencies have endorsed *in silico* strategies as valuable complementary tools for defining randomized clinical trials (RCTs), enhancing study design, and even circumventing certain studies in specific situations, such as drug repositioning (39). In this regard, over the past few decades, substantial progress has been made in collaboration with the pharmaceutical industry to develop good practice guidelines and recommendations for various computational approaches, including pharmacometrics models (e.g., for pharmacometrics models) (40, 41). However, the absence of established guidelines for modelling approaches in other disciplines, such as systems medicine, remains a notable gap (42). Nonetheless, there is a widespread consensus on the essential principles governing these approaches. For instance, the Good Practices in Model-Informed Drug Discovery and Development (MID3) describes the “quantitative framework for prediction and extrapolation, centered on knowledge and inference generated from integrated models of compound, mechanism, and disease level data and aimed at improving the quality, efficiency, and cost-effectiveness of decision making” (43). These guidelines also classify the evidence extracted from the modelling approaches in three categories based on their purposes and their impact for industry decision-making (44) or for regulatory assessment (45): “LOW” impact, when the evidence generated does not allow to make clinical or commercial decisions; “MEDIUM” impact, when the obtained data could be useful in strategic conditioning of future trial or experimental design; and “HIGH” impact, when conclusions obtained from modelling directly support decision-making without the need for additional experimental or trial studies (43).

We recently described a computational method, according to the above-mentioned guidelines, that combined different modelling approaches (virtual population [vPop] randomization through population deconvolution, physiologically based pharmacokinetic [PBPK] modelling, and systems biology [SB] modelling) to build quantitative systems pharmacology (QSP) models and simulate the mechanism of action (MoA) of a drug in a virtual patient population (46). Herein, our objective is to assess the potential of this approach in offering molecular insights into the drug’s MoA, thereby establishing mechanistic profiles through the evaluation of patient-specific archetypes. To investigate the method’s ability to explore response variability beyond a mere dose effect, we utilized psoriasis as a representative example of a complex and heterogeneous disease, along with the evaluation of two approved CZP dosing schemes. Thus, we modified and adapted this methodology to simulate the two officially approved CZP dosing regimens within a virtual population (vPop) with moderate-to-severe psoriasis. Our aim was to assess the method’s capability to accurately replicate non-standard demographic distributions (i.e., increasing body mass index [BMI]), and effectively model a non-small molecule compound at both the physiologically-based pharmacokinetic (PBPK) and quantitative systems pharmacology (QSP) levels. Subsequently, we conducted an extensive evaluation of the models using biological and clinical data, and employed clustering analyses to investigate the molecular variability captured by the models.

## 2 Methods

**Figure 1:**
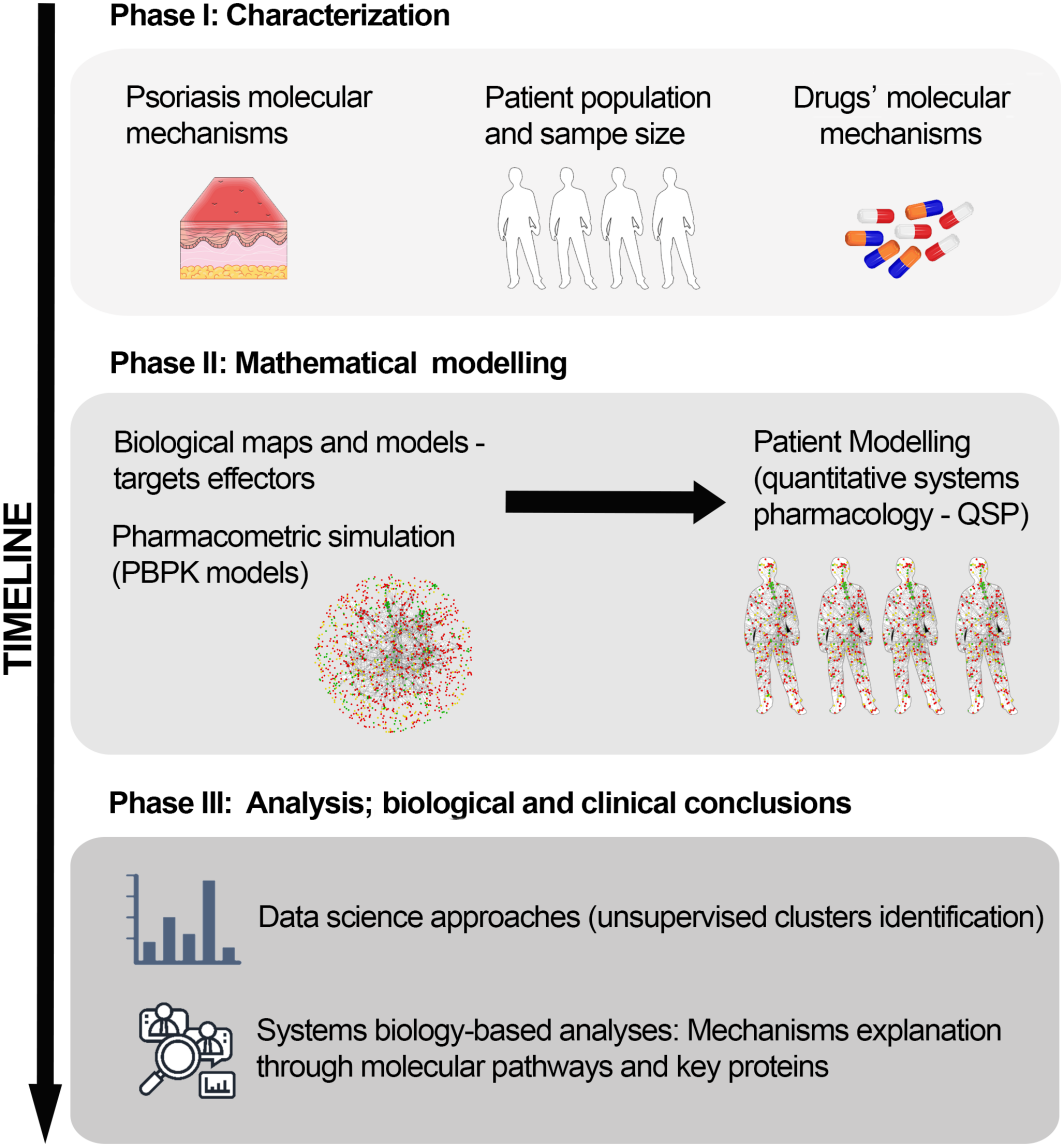
*In silico* clinical trial protocol overview. The protocol was divided into three main stages: Phase I, study design and information compilation; Phase II, mathematical modelling; and Phase III, data analysis to obtain molecular insights on the drug’s mechanisms of action. PBPK, Physiologically based pharmacokinetic; QSP, Quantitative systems pharmacology.

Combining several modelling approaches, we followed sequential stages to generate the QSP model set and to create the virtual patient population based on a RCT population (**Figure 1**). In the design phase, we compiled information and defined the population (condition and patient population characterization and sample size calculation) and intervention (drug characterization) details. In the modelling phase, we embedded a series of vPop, PBPK, and SB-based models (Therapeutic Performance Mapping System [TPMS]) to create QSP models. We exploited clinical efficacy information on drugs used in moderate-to-severe psoriasis, along with known molecular information on psoriasis severity, as prior information to generate the models. Finally, we performed SB-based analyses and examined the molecular variability among the virtual patients’ models by applying a comprehensive and robust clustering approach.

### 2.1 Population definition

#### 2.1.1 Demographical and clinical definition

To reflect real demographic and comorbidity parameters, we used psoriasis-related studies as a reference. We obtained population demographic information from the CIMPASI I, CIMPASI II and CIMPACT trials (23–25), while we inferred the frequency of comorbidities (diabetes, hypertension, non-alcoholic fatty liver [NAFLD], anxiety, and depression) from the prevalence and odds ratio in the population with psoriasis (47–50). For psoriatic arthritis, we used the self-reported (not diagnosed-based) frequency reported in the CIMPASI I, CIMPASI II and CIMPACT trials (23–25).

#### 2.1.2 Molecular definition

To characterize the populations’ disease and comorbidities in detail, we carried out a manual literature curation protocol as previously described (51, 52). For psoriasis definition, we initiated an extensive and careful full-length review of relevant articles found in the PubMed database up until the moment of the start of the study (restricted from October 2013 to October 2018) obtained by the following search string: *psoriasis [TITLE] AND (molecular [TITLE/ABSTRACT] AND (pathophysiology [TITLE/ABSTRACT] OR pathogenesis [TITLE/ABSTRACT]))*. We retrieved the list of publications identified and assessed them at the title and abstract level. When we found molecular information describing pathophysiology conditions, we thoroughly reviewed the full text to identify the main pathophysiological processes described as being involved in psoriasis (**Supplementary Table A** in the **S2 File**). We further characterized each pathophysiological process at the protein level by using the retrieved publications and saved for analysis all proteins whose activity (or lack thereof) was functionally associated with the development of the condition (**Supplementary Table A** in the **S2 File**). We characterized comorbidities with the same methodology.

Besides the bibliographical characterization, we collected additional data to further characterize moderate-to-severe psoriasis. We retrieved expression data from the Gene Expression Omnibus public repository (53) using the query [19^th^ November 2018]: psoria* [TITLE]. We only considered studies performed in humans through expression array (*Series type: Expression profiling by array*) with more than 30 samples. We obtained three datasets that fitted the mentioned characteristics: GSE13355 (54, 55), GSE14905 (56), and GSE78097 (57) (which included information on severity; we considered psoriasis as moderate-to-severe when the Psoriasis Area Severity Index [PASI] >=12). We analyzed experiments using GEO2R software with the default settings. We then selected the proteins that resulted positively or negatively differential between severe and mild psoriasis lesions (GSE78097; adj. P-value< 0.01, |log2FC|>1) and between psoriatic lesions and control biopsies in at least one of the two psoriasis vs. control experiments (GSE13355, GSE14905; adj. P-value< 0.01). We detected 58 genes as representatives for the severe psoriatic state with respect to mild lesions and control biopsies (**Supplementary Table B** in the **S2 File**).

#### 2.1.3 Sample size calculation

We calculated the minimum sample size of virtual patients to be generated as the population number for which a molecular classifier was able to discriminate between patients with psoriasis and healthy individuals while having enough statistical power. Given that TPMS models’ outcomes are based on predicted protein activity, we considered experimental measures that could relate to protein activity variability, particularly gene expression. We queried the Gene Expression Omnibus database (53) (February 2021) and obtained the following series containing the raw microarray files for skin biopsies from patients with psoriasis and controls: GSE13355, GSE14905, GSE78097 (**Supplementary Table C** in the **S2 File**). They totaled 209 patients (118 cases and 91 controls). We normalized raw data by using the CuBlock cross-platform normalization method (58) to enable analyzing experimental data from different platforms.

To identify the minimum sample size, we applied a previously described approach (46) based on a progressive sampling method (taking subsets from 10 to the maximum number of samples) proposed by Mukherjee et al. (59) and Figueroa et al. (60). This approach aims at identifying the minimum sample size needed to obtain a two-feature based classifier that achieves a determined discrimination accuracy—namely the maximum accuracy (“Max accuracy”) when considering all available samples (209 samples)—by applying a classification training procedure. We used K-fold cross-validated accuracy (k = 10) to validate the classifiers’ performance. The percentage of Max accuracy reached for each subset of samples and for the total was calculated using the classifiers obtained for that subset (**Supplementary Figure S1** in the **S1 File**). All tested subsets with 20 or more samples reached a 90% Max accuracy when computed with statistical powers of 95% (and even 99%). Considering statistical powers of 95%, the 95% Max accuracy was achieved with around 30 patients (i.e., 15 patients per cohort) and remained above the 95% Max accuracy threshold for the evaluated increasing set sizes. To have enough representativity of all comorbidity groups (the lowest in frequency incidence, diabetes type II - 0.438 (49), would need a population of 343 individuals to reach a cohort of 15 patients), and given that our computational approach allowed us to obtain patients more easily than RCTs, we established 500 virtual patients as the sample size.

#### 2.1.4 Virtual population simulation

For the present study, we generated a vPop resembling a real CT patient population with psoriasis since the patients with psoriasis CT populations differ from global population distributions by reason of its increased BMI and weight. We used the CIMPASI I, CIMPASI II and CIMPACT trials (23–25) to gather the reference distribution parameters. We retrieved age, weight, and BMI and constructed the vPop using an adapted version of the algorithm proposed by Allen et al. (61), as described elsewhere (46). We assumed that height was unrelated to the disease and used general distributions from European standard population values (62).

Additionally, we assigned molecular tags to the patients to account for comorbidity prevalence. Tags were distributed randomly among the virtual patients, with no co-occurrence frequency for any paired comorbidity. As an exception, we only assigned the *obese* molecular definition tag to patients presenting a BMI >30 kg/m^2^.

While demographic parameters were later used to obtain individualized PBPK models of the two CZP dosing schemes, we used comorbidity data, once translated into molecular information, as preliminary restriction information for generating the QSP models.

### 2.2 Intervention definition

In our model, the vPop was treated with the two approved dosing schemes of CZP. We used a two-branch scheme, one per dosage, to compare their differences, mimicking the CIMPASI I, CIMPASI II and CIMPACT trials. We used the same 500 virtual patients (i.e., same individualized PBPK models) to obtain the two study branches: subcutaneous administration of CZP 200 mg every two weeks for 16 weeks and subcutaneous administration of CZP 400 mg every two weeks for 16 weeks, with both arms receiving the loading dose of CZP 400 mg at weeks 0, 2, and 4. This resulted in two interventions, or QSP models, per patient, generating a total of 1,000 models.

#### 2.2.1 Certolizumab pegol molecular and pharmacokinetic characterization

To characterize CZP, aside from reviewing official regulatory documentation and drug-target dedicated databases, we performed an evaluation of the currently available bibliography regarding known targets of the drug, as well as pharmacokinetic information, in PubMed (search restricted from October 2013 to October 2018). The specific search queries were the following: certolizumab [TITLE] AND (target [TITLE/ABSTRACT] OR molecular [TITLE/ABSTRACT] OR pharmacokinet* [TITLE/ABSTRACT]). We analyzed all articles at the title and abstract levels. From those selected, we reviewed the presence of molecular and pharmacokinetic information in depth to identify possible proteins/genes to be considered drug target candidates, as well as additional information for subsequent modelling (**Supplementary Table D** in the **S2 File**).

#### 2.2.2 Physiologically based pharmacokinetic modelling

To assess the relation of each dosage with the drug’s concentration in body organs, we built a PBPK model for each virtual patient. Because antibodies primarily distribute within the blood system, with low penetration in other organs and tissues, we constructed a two-compartment model system consisting of only the skin (administration tissue) and the blood (clearance system). We applied the equations associated with blood flow rates and skin volumes described by Brochot and Quindroit (63). However, blood volume was adjusted to fit the drug’s distribution volume for optimized modelling. Since these variables depend on cardiac frequency, age, BMI, and sex, they yielded individualized models as described elsewhere (64).

The absorption constant (*k_a_*) was calculated using the following formula (65):


(Equation 1) ka=ln2T12a

Where T12a is the absorption half-life.

When approximating T12a≈Tmax/3 (66), we obtained:


(Equation 2) $$k_{a}=\frac{\left(ln2\right)}{{\text{T}}_{max}/3}$$

The clearance constant parameter (*k_el_*) was calculated by fitting a general model to pharmacokinetics data points. We used European Medicine Agency’s C*_max_* values, and additional points were extrapolated using C*_max_*, half-life, and T*_max_* parameters (67, 68).

We implemented all PBPK compartment models in MATLAB™ (69) and integrated differential equations describing the kinetics of the compounds and the fitting procedures by using the SimBiology Toolkit.

### 2.3 QSP modelling – obtaining virtual patients

#### 2.3.1 Systems biology-based modelling – TPMS

TPMS technology (51) generates mathematical models by applying supervised machine learning methods based on a human protein functional network, using known biological, medical, and pharmacological information as training data (**Supplementary Table E** in the **S2 File**). These models can be used to simulate the behavior of drugs and the pathophysiology of diseases in terms of changes in protein activity (52, 70–72).

Here, we used TPMS to build the mathematical models to simulate the behavior of CZP over psoriasis by modelling the changes in disease-related proteins’ activity (**Supplementary Table A** in the **S2 File**). Besides TPMS training data, which included specific psoriasis treating and inducing/exacerbating drugs (**Supplementary Table E** in the **S2 File**), we used additional molecular information to denote different patient types, including (i) gene expression in moderate-to-severe psoriasis (**Supplementary Table B** in the **S2 File**) and (ii) psoriasis common comorbidities (diabetes, hypertension, NAFLD, anxiety, and depression).

TPMS mechanism of action models can be defined by the predicted protein activity achieved for each protein (ranging between -1 [completely inhibited] and 1 [completely activated]) by the flow of the signal through the protein-protein interaction network (51). From the predicted protein activity of the proteins designated to define psoriasis, we calculated the previously described TPMS model-derived parameter tSignal (Equation 3) (51), which ranges between -1 and 1, and used it for the molecular definition of psoriasis (**Supplementary Table A** in the **S2 File**) in the QSP models:


(Equation 3) $$tSignal=\frac{1}{n}\sum_{i=1}^{n}v_{i}y_{i}$$

Where *n* is the number of proteins defining the protein set with non-zero signal; *v_i_* are the protein signs (active or inactive) according to each disease/comorbidity definition; and *y_i_* are the resulting modelled signal values achieved by each protein “*i*” after stimulating the model with the corresponding drug.

#### 2.3.2 Integrating PBPK and systems biology modelling – QSP models

We computed QSP models for each patient-dosage using the TPMS methodology as previously described (46). Briefly, we used two types of restrictions to generate the models: (i) quantitative dose-related data, which are related to the effectiveness of the drug-dosage, and was computed using concentration data and effectivity relation; and (ii) molecular data, which can be subdivided in patient-specific molecular information related to his/her disease and comorbidity characterizations, and protein known information from publicly available databases and Biological Effector Database (51). Consequently, the resulting models allowed evaluating the influence of the different dosing schemes.

In order to link the CZP concentration with efficacy, a proxy model-derived EC50 parameter was calculated as described previously (46). Here, a set of clinical trials evaluating moderate to severe psoriasis using different drugs were included with PASI75 as outcome measure, which were modelled using the same PBPK modelling strategy as for CZP (efficacy data, specific PK parameters and data sources in **Supplementary Table D** in the **S2 file**). Exceptionally, infliximab, however, was modelled as a single compartment, with an intravenous dosage; and for apremilast a model previously described (46) was used, with an oral dose and liver clearance.

### 2.4 Data analysis

#### 2.4.1 Statistical treatment

We analyzed the generated data with MATLAB™ functions and Python or R packages. For the analysis of the population demographic and molecular parameters, we used descriptive statistics (mean and standard deviation, frequency tables, or pie charts) and applied the appropriate parametric and non-parametric tests. We used Pearson’s correlation to evaluate the fitting of tSignal to clinical and severity measures. We used Wilcoxon rank-sum test for the comparison of predicted protein activity level. In all cases, we reported the applied test and calculated the false discovery rate (FDR) according to Benjamini-Hochberg (73) multi-test correction method to control for type I errors, whenever applicable. We set the statistical significance level based on p- or q-values for each analysis, always being, at least,<0.05. We applied a data science-based approach (51) to identify classifier molecules by using cross-validated accuracy as a quality measure.

A selection and conversion methodology was applied (46) to evaluate whether the predicted protein activity of the proteins defined in the psoriasis definition were able to fit an efficacy metric, namely PASI75, as defined in available clinical trials (**Supplementary Table D** in the **S2 file**). We evaluated the tSignal on the set of drug models created as a proxy of this clinical efficacy measure. In order to link the clinical efficacy measure, PASI75, with the model-derived value, ADHD-tSignal, linear regression analysis (Pearson’s correlation) between both variables was performed to parameterize the following equation:


(Equation 4) Clinical efficacy measure=A*model−derived efficacy+B

This process was designed to maximize the absolute value of the Pearson correlation coefficient (|ρ|) between clinical and tSignal values, maintaining molecular information from the bibliography-based characterization. Proteins within the psoriasis molecular definition distorting this relationship were identified and discarded iteratively to optimise the correlation and identify the protein set that best fitted the clinical measurements.We calculated the accuracies of SB and QSP models for each solution within each model and expressed them as the percentage of compliance of all drug-pathophysiology relationships included in the training set (51).

#### 2.4.2 Clustering algorithms

We used an unsupervised clustering strategy to find molecular patterns in the resulting model’s output. As input data, we used the resulting signal values of psoriasis’ characterized proteins for all patient-dosage models. First, we carried out a feature (protein) normalization for each arm by subtracting the mean predicted protein activity value to minimize the potential direct impact of drug concentration per patient without compromising inter- and intra-protein variability. Then, we applied a dimensional reduction method using Principal Component Analysis (74) or Multidimensional Scaling (MDS) (75) with two, three, and five dimensions. We obtained the clusters by applying a set of different clustering algorithms: Kmeans (76), self-organizing map (77), Spectral (78), Gaussian mixture model (79), and hierarchical (80). We encapsulated all the methodology in a single clustering strategy analysis, which also computed the optimal number of clusters for each setting, defined according to Calinski Harabasz (81), Davies-Bolduin (82), Gap (83), and Silhouette (84) indexes, being the latter prioritized among the rest. We evaluated the quality of the resulting clustering analyses by three quality indicators: Hopkins statistics (to measure the clustering tendency of a data set) (85), Dunn index (to evaluate the cluster compaction) (86), and Jaccard Bootstrap Index (to measure the stability of the clustering solution in a set of bootstrap resamples) (87). Once the best model was identified, we computed the Euclidean distance between clusters to evaluate their proximity.

#### 2.4.3 Enrichment analysis

To functionally evaluate the protein predicted activity, we carried out a hypergeometric enrichment analysis (88) over the proteins with a more characteristic behavior in each cluster compared with the complete set of model solutions. The complete list of proteins included in the models was used as protein universe. We used KEGG (89), Gene Ontology (90), and TRRUST (91, 92) as reference databases. We only selected enriched pathways with an FDR q-value< 0.01. Moreover, we excluded those containing either more than 300 genes—to keep biologically specific results—or less than 10 genes—to reduce artifacts. We used a modification of Hausdorff distance (93) between the enriched sets over the human protein network (51) as a link value between the sets to represent the results’ network. We performed all network representations with the software Cytoscape (94).

### 2.5 Computational resources

We executed all simulations described in this study in the Anaxomics’ cloud computing server, which integrates more than 800 computational threads in machines with 64 Gigabytes of RAM. Software, databases, and tools are the property of Anaxomics Biotech.

## 3 Results

### 3.1 Virtual population distribution matched literature patient demographics

**Figure 2:**
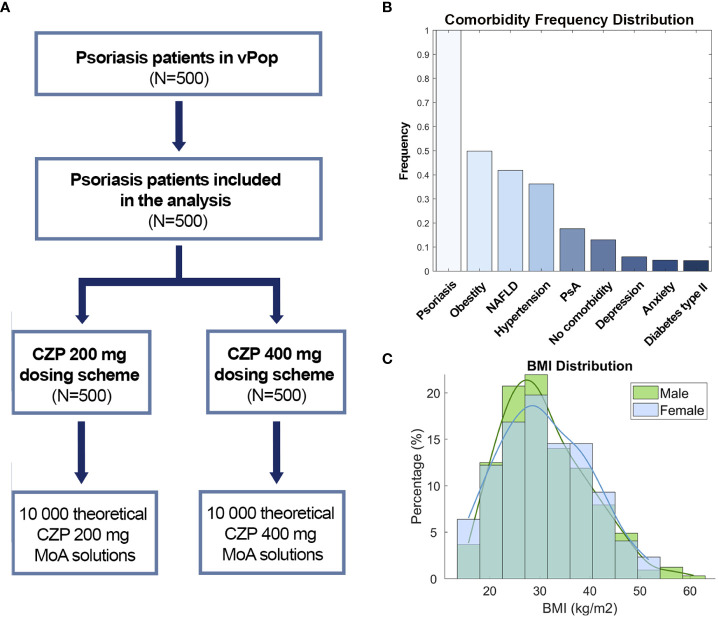
**(A)** Study branch scheme, **(B)** Comorbidity frequency distribution and **(C)** BMI distribution by sex. CZP, Certolizumab pegol; NAFLD, Non-alcoholic fatty liver disease; PsA, Psoriatic arthritis; BMI, Body mass index.

According to statistical evaluation, the patient deconvolution and vPop simulation led to the generation of a vPop with moderate-to-severe psoriasis that reproduced the demographic characteristics of real clinical trial reference populations (no statistically significant differences with the mean values of clinical trials, **Table 1**). As expected, mean weight and BMI were found higher than in general populations, with high proportions of patients with overweight - BMI >= 25 - and obesity - BMI >= 30 (**Figure 2C**). When comparing our vPop o a European-like population (62) with the same population size, age distribution, and sex ratio to assess demographic distribution similarity, both weight and BMI were found to be significantly higher in the vPop with psoriasis than in the European population (Wilcoxon rank sum test, p< 0.001). No differences were observed for height (Wilcoxon rank sum test, p= 0.802) or for age (Wilcoxon rank sum test, p= 0.226). Additionally, the vPop was created to include comorbid patients according to real-world data (**Figure 2B**), achieving a population of 87% moderate-to-severe psoriasis patients suffering at least one comorbidity. The 500 virtual patients, defined by their characteristics, were submitted to PBPK and SB mechanistic modelling of the two dosage regimens of CZP (**Figure 2A**), obtaining two arms of models for the same patients.

**Table 1:** Demographic characteristics of the generated virtual population and the reference population (23–25).

	Virtual population (N=500)	Reference population^a^ (N=1,020)	p-value^b^
Sex (% females)	34.4	34.4	NA
Age (years)	46.03 ± 14.76	45.53 ± 13.23	0.40
Height (cm)	170.67 ± 12.54	-^c^	NA^c^
Weight (kg)	89.58 ± 23.87	90.73 ± 22.71	0.26
BMI (kg/m^2^)	30.99 ± 8.68	30.48 ± 7.07	0.11

Values are mean (± standard deviation) unless otherwise stated. Boxplot representations can be found in **Supplementary Figure S2** in **S1 File**.

BMI, Body mass index; NA, Not applicable.

a Values taken from the CIMPASI I, CIMPASI II and CIMPACT clinical trials.

b Calculated with the unpaired two-tailed Student’s T-test.

c For demographic data not provided in the clinical trials, European mean values were used for modelling.

### 3.2 Physiologically based pharmacokinetic models fitted literature data

**Figure 3:**
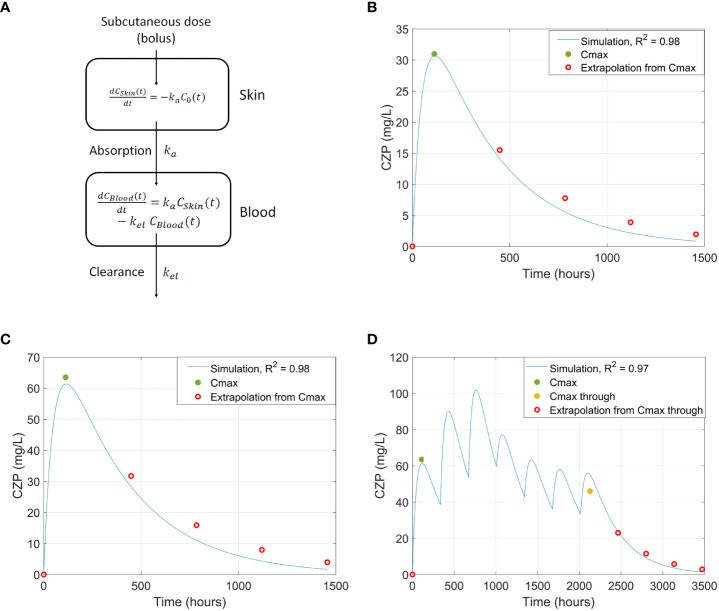
**(A)** PBPK model representation. **(B-D)** Comparison of CZP concentration in blood between literature’s Cmax values and extrapolated datapoints and the simulated curve using the PBPK model, generated from a standard adult patient, for: **(B)** 200 mg single dose of CZP (67); **(C)** 400mg single dose of CZP (67); and **(D)** 3 x 400 mg loading doses + 4 x 200mg doses (95). CZP, Certolizumab pegol; PBPK, Physiologically based pharmacokinetic.

Several Cmax concentration datapoints were used to fit the PBPK models: 200 mg dose Cmax, 400 mg dose Cmax (67), and 400 mg initial dose followed by 200 mg doses until achieving Cmax at the steady state (95). Additionally, clearance-related datapoints were extrapolated and used to refine the model (68). The resulting PBPK models adjusted to all reported Cmax values and extrapolated clearance data points with an R^2^ > 0.95 (**Figure 3**). The calculated Tmax, half-life, and Cmax parameters for the standard-patient (75 kg, 170 cm, 40 years old) model were similar to literature values (**Supplementary Table F** in the **S2 file**).

### 3.3 SB and QSP models were able to reproduce known biological and clinical activity

The MoA of CZP in our vPop (QSP models) presented a mean accuracy value of 91.51% with respect to the training information. These simulations took into account the whole available data on pathologies, drugs, and the populations’ characteristics. Once built, the potential of QSP models to reproduce clinical and molecular data was tested.

We evaluated the psoriasis tSignal in models developed for the different psoriasis drugs to assess the correlation between the literature-based psoriasis definition and clinical observations (i.e., PASI75 scores). We identified a list of 115 proteins out of the 124 original psoriasis protein effectors (**Supplementary Table A** in **S2 File**) that, when measured together using the tSignal, correlated with |ρ|=0.85 to PASI75 measurements of (**Supplementary Figure S3** in **S1 File**).

We analyzed the tSignal calculated from the optimized psoriasis definition of the CZP models developed for the virtual patient population. The tSignal was then compared with the predicted protein activity of known psoriasis severity-associated proteins. The predicted protein activity of 12 proteins were identified to correlate to the TPMS-psoriasis tSignal, 9 of them presenting moderate correlation (|ρ|>0.5) for both dosing schemes (**Table 2**).

**Table 2:** Evaluation of correlation of predicted protein activity of proteins previously shown to correlate with psoriasis clinical severity (measured according to PASI) with tSignal.

Gene name	UniProt code	CZP 200 mg		CZP 400 mg		Reference to psoriasis severity
		Rho (ρ)^a^	FDR q value^a^	Rho (ρ)^a^	FDR q value^a^	
IFNG	P01579	-0.77	4.80E-97	-0.75	8.06E-90	(96)
S100A9	P06702	-0.76	1.50E-92	-0.75	7.88E-93	(97–99)
FLT1	P17948	-0.68	1.07E-67	-0.70	8.54E-74	(100)
VEGFA	P15692	-0.66	5.82E-64	-0.63	1.46E-57	(100–103)
NAMPT	P43490	-0.65	1.82E-59	-0.63	1.16E-55	(104)
SLC2A1	P11166	-0.64	3.11E-58	-0.56	3.83E-42	(105)
PRL	P01236	-0.57	4.28E-43	-0.56	3.20E-42	(106)
MMP9	P14780	-0.54	1.47E-38	-0.66	4.75E-65	(107)
CRP	P02741	-0.53	1.06E-36	-0.55	4.06E-40	(108–111)
IL1B	P01584	-0.47	1.36E-28	-0.51	9.23E-34	(112, 113)
LEP	P41159	-0.43	8.75E-24	-0.36	3.55E-17	(114)
TNF	P01375	-0.33	5.79E-14	-0.64	6.52E-59	(112, 115, 116)

Calculated for the two dosing schemes (CZP 200 mg and 400 mg).

CZP, Certolizumab pegol; FDR, False Discovery Rate; PASI, Psoriasis Area Severity Index.

a Pearson’s correlation Rho and the respective adjusted p-value are indicated. Only results with non-negligible correlation are presented (|Rho|>0.3).

Grey-shaded genes show strong or moderate correlation to tSignal for both dosing schemes. Correlation strength: strong – |rho|>0.8; moderate – 0.8>|rho|>0.5; low – 0.5>|rho|>0.3; negligible – |rho|<0.3.

We observed a strong correlation between the optimized psoriasis tSignal measure and the original psoriasis tSignal (i.e. considering the complete response) within the CZP models in the virtual patients population (ρ>0.99 for both dosing schemes).

### 3.4 Patient-specific QSP mechanistic models can be clustered according to their molecular variability

**Figure 4:**
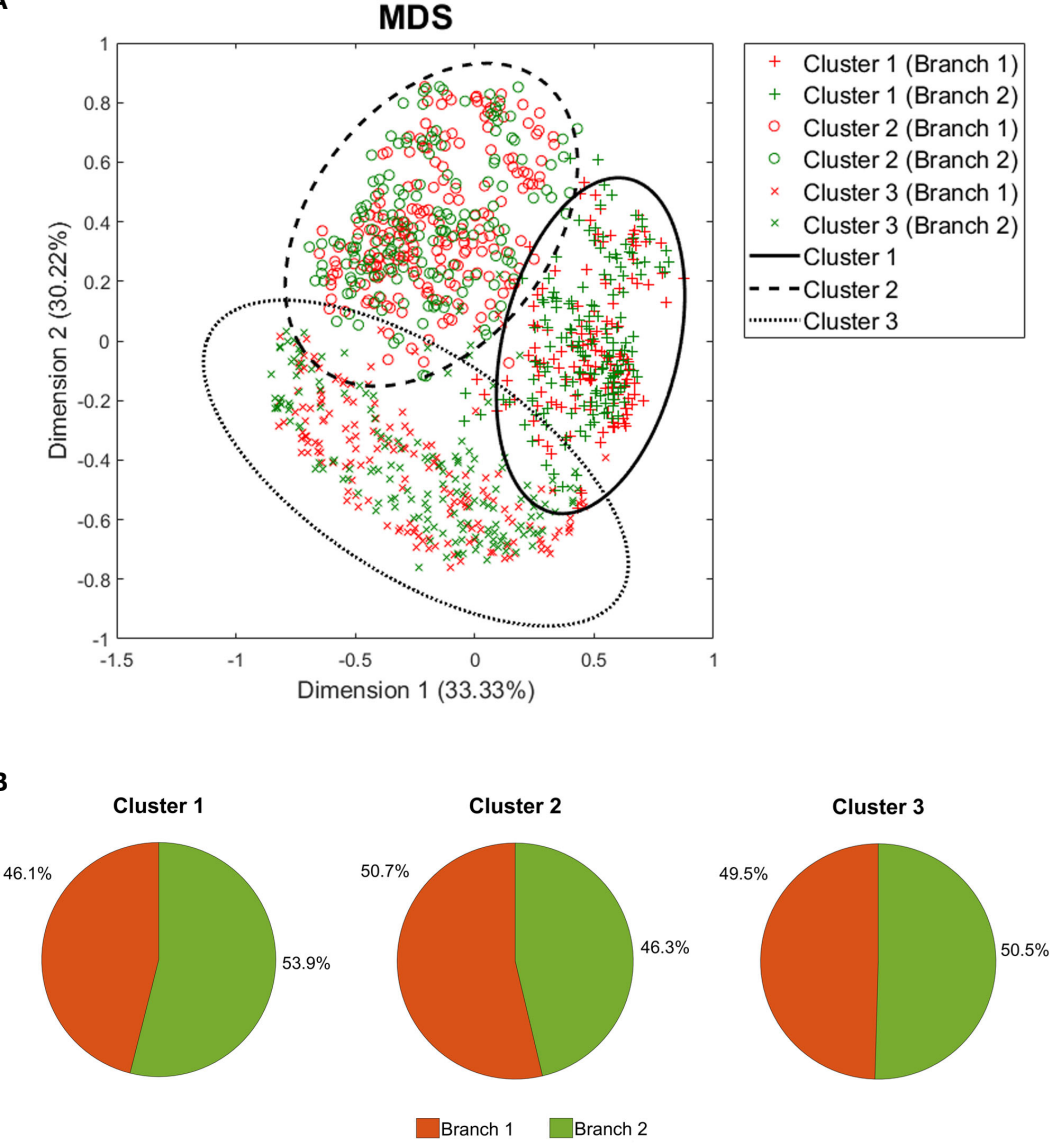
Clustering analysis results. **(A)** Two-dimension representation of the best clustering setting result using all 1,000 patient models after MDS, and **(B)** the branch incidence of each obtained cluster. MDS, Multidimensional scaling.

To identify distinct mechanistic response patterns to CZP, we performed clustering analysis on the individualized patient models’ response to the drug using the predicted protein activity as a measure of variability. Several combinations of dimensionality reduction and clustering algorithms were applied, and the best model according to Hopkins (0.84), Dunn (0.09), and Jaccard Bootstrap (0.53) indexes was selected. The resulting model involved a hierarchical clustering algorithm with Euclidean distance and average aggregation function, using a five dimensions reduction space after MDS with Spearman coefficient as distance. This model identified three mechanistic clusters as optimal (**Figure 4A**) with a similar proportion of patients from both dosage arms (**Figure 4B**). Clusters were represented using the two main dimensions of MDS (**Figure 4A**), which, combined, explained the observed variability of 63.55%. According to Euclidean distance, all three clusters were at a similar distance from each other, with cluster 3 being the most distant among them (**Supplementary Table G** in the **S2 file**).

Clustering was found to be associated with differences in the dose-normalized tSignal. Cluster 1 showed a higher signal (with respect to the mean signal for the entire population per branch) than the rest of the samples (Student’s T-test, p< 0.001), while cluster 2 presented the lowest value (Student’s T-test, p< 0.001). To further characterize the clusters, additional comparison analyses were performed to identify potential differences regarding the demographic characteristics within the clusters. When comparing each cluster against the rest, cluster 1 was found to accumulate the highest ratio of females (Chi-squared test, p< 0.001) and the lowest height and weight values (Student’s T-test p< 0.001). On the other hand, cluster 2 presented a higher percentage of males and the highest values for weight and BMI (Student’s T-test p< 0.001) (**Table 3**).

**Table 3:** Results of the comparison analysis between demographic characteristics within the clusters.

ID	Age, *years*	Height, *cm*	Weight, *kg*	BMI, *kg/m^2^*	Sex, *M:F ratio*
1	46.46 ± 15.25 (0.537)	161.63 ± 10.85 (**<0.001**)	78.00 ± 23.23 (**<0.001**)	30.41± 10.37 (0.165)	0.39 (**<0.001**)
2	45.88 ± 14.18 (0.814)	177.45 ± 9.59 (**<0.001**)	101.83 ± 20.36 (**<0.001**)	32.61 ± 7.42 (**<0.001**)	0.83 (**<0.001**)
3	45.79 ± 14.79 (0.717)	171.51 ± 11.78 (0.131)	86.80 ± 21.75 (**0.009**)	29.75 ± 7.96 (**0.001**)	0.70 (**0.028**)

Figures are mean ± standard deviation (and p-values*).

*Student’s T-test: each cluster vs. rest of clusters.

BMI, Body mass index; ID, Cluster ID; M:F ratio, Male to female ratio. p-values in bold are those considered statistically significant (p< 0.05).

The proportion of patients suffering from comorbidities in each cluster was also evaluated and resulted in statistically significant findings. Cluster 2 showed a higher frequency of obese patients (Fisher test p< 0.001) than the other clusters. Conversely, cluster 1 had more patients suffering from diabetes type II and NAFLD (Fisher test p< 0.001) than the remaining clusters. Finally, cluster 3 stood out by accumulating all non-comorbid patients, comprising the lowest frequency of patients with diabetes type II, hypertension, NAFLD, and obesity (Fisher test p< 0.001).

**Figure 5:**
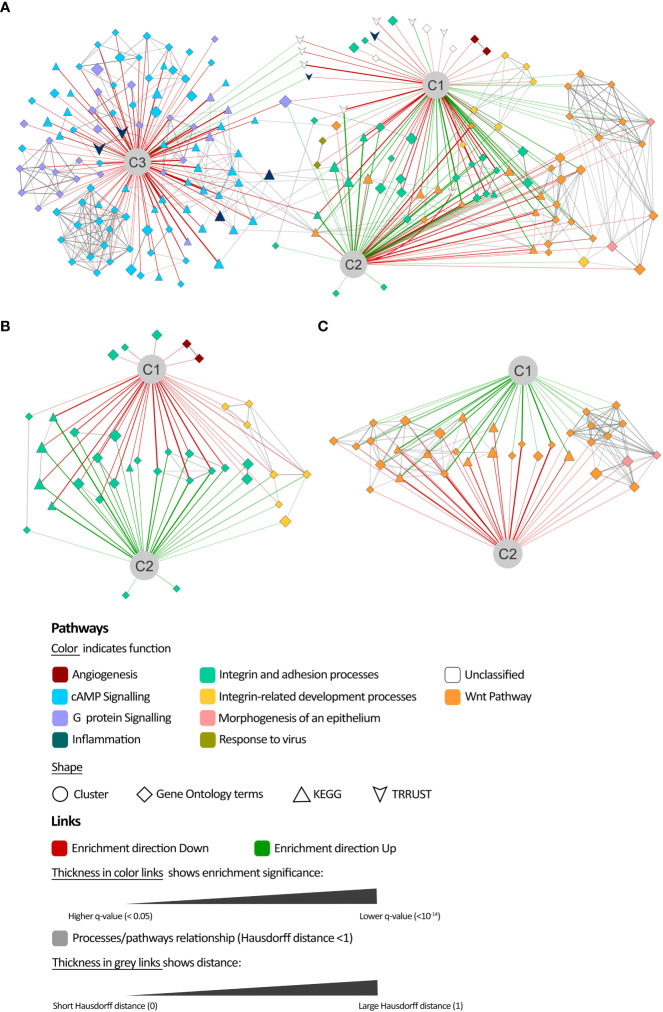
Comparison of mechanisms of action enrichment analysis. **(A)** Network representation of the enrichment analysis results of the most differentially modulated proteins in each cluster (C1, C2, and C3) with respect to the population mean and their relationship. Details on the least active processes in **(B)** cluster 1 and **(C)** cluster 2.

To elucidate the underlying processes contributing to the mechanistic differences between clusters, enrichment analyses were conducted for the proteins from all models that differed in their mean predicted activity value more than 0.1 from the overall value in each cluster (Wilcoxon rank-sum test, FDR q< 10E-4, **Supplementary Table H** in the **S2 File**) (**Figure 5**, **Supplementary Table I** in the **S2 File**). In order to consider the direction of the differences, separate enrichment analyses were conducted for proteins exhibiting higher activity (UP) and lower activity (DOWN) within in each cluster, as compared to the overall model solutions (**Supplementary Table H** in the **S2 File**). According to the distance analysis, cluster 3 shared only a few enriched processes with clusters 1 or 2, which were related to signaling by cAMP and G proteins (**Figure 5A**). Clusters 1 and 2 demonstrated a similar profile of processes, although with an opposite modulation tendency compared to the mean population. Within the least active processes in cluster 1 (**Figure 5B**), the main components were integrin and adhesion-related pathways, including processes related to early development (*formation of primary germ layer*, *gastrulation, embryonic morphogenesis, endoderm formation, endoderm development, endodermal cell differentiation, mesodermal cell differentiation*). Angiogenesis-related pathways (*positive regulation of angiogenesis, positive regulation of vasculature development*) were also found among the least active processes in cluster 1. On the contrary, Wnt-related pathways were the least active in cluster 2 (**Figure 5C**) and included epithelial formation pathways (*morphogenesis of epithelium, morphogenesis of embryonic epithelium*).

By applying feature selection and classification algorithms, we successfully identified proteins whose predicted activity allowed the differentiation of mechanisms within each cluster with a cross-validated balanced accuracy of at least 0.8 (**Table 4**).

**Table 4:** Psoriasis effectors that best classify the patients in each cluster.

Gene name	UniProt code	Mean normalized predicted protein activity value			BACC		
		C1	C2	C3	C1 vs C2	C1 vs C3	C2 vs C3
ITGB1	P05556	-0.15	0.19	-0.07	**0.964**	0.662	**0.841**
ITGAE	P38570	-0.12	0.09	0.01	**0.966**	0.780	0.702
ITGA1	P56199	-0.18	0.24	-0.09	**0.968**	0.635	**0.845**
KDR	P35968	-0.06	0.07	-0.02	**0.928**	0.685	0.764
S100A9	P06702	-0.17	0.04	0.11	**0.923**	**0.913**	0.653
ITGB7	P26010	-0.17	0.24	-0.11	**0.974**	0.623	**0.877**
ERAP1	Q9NZ08	0.06	0.05	-0.11	0.510	**0.958**	**0.959**
IL4	P05112	-0.10	-0.01	0.11	0.667	**0.885**	**0.809**
DEFB4A	O15263	0.08	0.09	-0.18	0.526	**0.902**	**0.919**
FLT4	P35916	-0.01	0.01	-0.01	**0.897**	0.587	**0.840**
CXCL8	P10145	-0.19	0.03	0.14	**0.811**	**0.868**	0.645
FLT1	P17948	-0.03	0.04	-0.01	**0.890**	0.620	0.798
IL18	Q14116	0.02	0.08	-0.10	0.599	**0.834**	**0.869**
CCL20	P78556	0.04	0.06	-0.10	0.543	**0.854**	**0.892**
ICAM1	P05362	-0.13	-0.05	0.17	0.633	**0.838**	0.799
PPARD	Q03181	-0.03	-0.03	0.07	0.561	**0.823**	**0.832**
IFNA2	P01563	-0.01	-0.01	0.03	0.526	**0.844**	**0.837**
DEFB103A	P81534	-0.02	-0.02	0.04	0.523	**0.837**	**0.823**
THBS1	P07996	0.02	0.02	-0.05	0.520	**0.810**	**0.829**
EFNA1	P20827	-0.02	-0.03	0.06	0.507	**0.819**	**0.819**

Only proteins showing a BACC> 0.8 (highlighted in bold font, cross-validated p< 0.001) to classify between, at least, two of the clusters are included. The mean normalized predicted protein activity value for each cluster is provided, and values with a difference of more than 0.1 in absolute value with respect to the population mean are highlighted. Values above the mean of the remaining solutions are shaded in green and those below in red.

BACC, Cross-validated balanced accuracy; C1, C2, and C3: Cluster 1, Cluster 2, and Cluster 3.

## 4 Discussion

In the present study, we applied an *in silico* technology to build a QSP model of a clinical trial-like vPop with moderate-to-severe psoriasis treated with two dosing schemes of CZP. We successfully applied and combined different modelling approaches, namely: (i) an algorithm to generate vPop in accordance to RCT reference population values and comorbidity frequencies in real-world populations; (ii) PBPK models of the dosing schemes of CZP in each virtual patient; and (iii) SB-based models of the MoA of the drug, which provided outcomes that correlated with clinical efficacy measures and previously reported molecular markers of psoriasis severity (e.g. PASI score). Furthermore, we were able to identify clusters of virtual patients based on their psoriasis-related protein predicted activity when treated with CZP, which could help unravel differences in the drug efficacy in diverse subpopulations, classified according to the proteins involved in the disease’s MoA.

Utilizing epidemiological data, we successfully constructed a virtual population (vPop) that closely resembled a randomized clinical trial (RCT) population in terms of demographic parameters and frequencies of comorbidities (23–25, 47–50). Although the vPop compiled the anticipated demographic parameters and comorbidity frequencies, the relationship between these parameters was not taken into account. For instance, while patients were allowed to have up to two or three comorbidities, and obesity was assigned to individuals with a BMI>30, potential associations between demographic characteristics and comorbidities beyond these specific criteria were not considered. Indeed, we obtained a 13% of non-comorbid patients with psoriasis; this figure could not be verified because of the lack of epidemiological information on psoriasis and overall comorbidity. Moreover, it is possible that this percentage might be underestimated due to the scarcity of patients with multiple comorbidities in the dataset. In the same line, the number of obese patients included was based on the BMI distribution of the reference clinical trials (23–25), yielding 50% of obese patients in our study. This number was higher than the frequency reported on the clinical trials (around 40%), or the one that could be inferred from epidemiological data on obesity and psoriasis (around 29%) (12, 117). Thus, our method created a population enriched in obese patients, while underestimating or neglecting the number of extremely obese patients in real settings. The limitations associated with the algorithm used to generate virtual populations may impact the accuracy of conclusions regarding the percentage of the population. However, these limitations do not affect the validity of comparisons between different pharmacological strategies within the same population (46).

Our PBPK and SB-based models demonstrated high accuracy in reproducing known data. Through the identification of a subset of psoriasis-related proteins within the literature-based psoriasis definition, we successfully established a correlation between clinical efficacy values from clinical trials testing psoriasis drugs (i.e., PASI75) and the model-derived psoriasis activity proxy [i.e., the tSignal (51)]. This subset contained the majority of the initially identified proteins, further validating the accuracy of our approach. When considering the CZP models in the virtual population, the tSignal considering both definitions (original and optimized) strongly correlated. In addition, the predicted activity of proteins known to correlate with disease severity (measured with PASI), was also found to correlate with the tSignal, including inflammatory proteins [IL-1β (118, 119), TNFα (112, 115, 116), CRP (109–111, 120)], proteins related to skin immunological barrier and epithelial hyperplasia [S1009 (121–125), IFN-γ (124, 126, 127)], angiogenic proteins [VEGF/FLT1 (128), MMP9 (129)], adipokines [NAMPT (104, 116)], leptin (114, 130, 131), sexual hormones [prolactin, previously associated to autoimmune diseases (132)], and a glucose metabolism protein related to both keratinocyte proliferation (133, 134) and T cell activity regulation (135, 136). These results suggested that our models, and our psoriasis definition, could be useful to answer drug efficacy questions.

In this sense, our observations of the unsupervised clustering of patients regarding their predicted mechanisms match with previously published data. Both our study and existing literature consistently show that large morphometric characteristics (weight, height, BMI), particularly obesity, are associated with a poorer response to anti-TNF treatment (137–139). In line with our results, age has not been found to affect the biologics’ effectiveness in psoriasis (140). Interestingly, our models indicated a better response to CZP in women, despite conflicting evidence in the literature regarding the impact of sex on treatment efficacy for CZP and the anti-TNF class (139, 141–148). Given that most previously published data focused on adalimumab treatment, with limited CZP data included, it remains unclear whether the observed sex differences are specific to CZP, the entire anti-TNF class, or influenced by biological or gender-based factors such as hormonal levels, immunological status, age of diagnosis, or access to treatment. They might also be explained by sex differences in comorbidities. However, our population did not specifically model the association between female sex, comorbidities, and their clinical manifestations. Nevertheless, the impact of sex on the response to CZP in psoriasis may involve complexities beyond drug concentration differences, which were not captured by our QSP models. This limitation highlights the need for further investigation. Our results on the impact of comorbidities, especially regarding cluster 3 grouping all patients with psoriasis and no comorbidities, suggested that co-occurrent comorbidities could result in mechanistic differential response to treatment, supporting the current tendency towards managing patients with a multi-disciplinary approach (16). Further molecular analysis of these models, beyond the scope of this study, could uncover relevant hypotheses in this regard.

The molecular evaluation of the clusters showed that there is a molecular diversity within the model solutions and virtual patients to CZP mechanism of action, and that the processes involved in this diversity are related to psoriasis development. Our results showed that CZP treatment could regulate integrin and adhesion-related processes associated with immune cells recruitment and homing (149), as well as keratinocyte function (150–152) and tissue differentiation. While anti-TNF treatment has been related to the modulation of inflammatory cell recruitment and homing, its effect on integrin regulation has not been well established. Since the regulation of integrins and their interaction partners is key for homeostasis of the skin (151), a detailed evaluation of these results is granted for understanding whether the downregulation of this activity could be relevant to CZP mechanistic efficacy. Our models highlight that modulating the Wnt pathway, involved in psoriasis development (153) through its role in hyperproliferation of keratinocytes and angiogenesis (154–156), could be related to anti-TNF efficacy. TNFα has been reported to reduce the canonical Wnt/β-catenin pathway through DKK-1 induction (157), linking inflammation, particularly TNF, to bone pathology (158). Anti-TNF treatment in rheumatic conditions, such as psoriatic arthritis and rheumatoid arthritis, has been found to modulate this pathway (159–162). Non-canonical Wnt signalling pathways have been recently highlighted as relevant in inflammatory disorders (e.g., psoriasis and psoriatic arthritis) (163, 164). The reduced activity of these pathways in cluster 2 seemed to indicate suboptimal TNF inhibition. These findings might pinpoint the importance of effectively controlling the inflammatory component of psoriasis to prevent the development of rheumatic complications, including psoriatic arthritis. Given that the identified clusters presented different tSignal values, these results could point to different psoriasis pathophysiological mechanisms or CZP therapeutic mechanisms that could be determinants of clinical efficacy, although prospective validation is required.

Our study can be framed within the increasing tendency to leverage recently available high-performance computing technologies in the field of biomedicine. The use of these technologies, combined with regulatory frameworks, will advance precision medicine pipelines, enabling personalized healthcare while reducing, refining, and partially substituting animal and human experimentation (165). According to the MID3 guidelines definitions, this theoretical model yielded conclusions classified as MEDIUM, suggesting its potential as a reliable hypothesis-generation tool capable of providing molecular insights. However, further experimental and clinical assays are necessary before its translation into clinical practice.

In our study, we generated subject-specific models from population information deconvolution. While the use of population aggregated values solves the potential ethical issues implied in managing individualized patient data (165), it entails the limitation of the patients not being real patients, precluding a conclusion for personalized medicine. As previously discussed, potential associations between the evaluated factors, impacting population frequencies and molecular/clinical differences, were not considered in our study (e.g. co-occurrence of comorbidities, increased risk for occurrence of comorbidities depending on the patient profile, or associations between patient characteristics not occurring in general population). Other factors, such as smoking or alcohol consumption, which are challenging to define at the molecular level, were neither taken into consideration. The use of demographic data and distributions from real-world psoriasis registries can help correct biases in RCT values, including those related to inclusion and exclusion criteria (e.g. exclusion of females of childbearing age). However, it is important to note that this approach has limitations in evaluating highly specific subpopulations and relies on the assumptions made for each profile’s modeling (165). This methodology can build true patient-specific models when the necessary information is available, such as through real-world data registries. With computational power as the only limitation, our approach allows for the recruitment of a great number of patients, which can be difficult, costly, and even not feasible in a conventional clinical trial setting. Moreover, paired analysis of the same subject in relation to different interventions, akin to a cross-over study, can be conducted without requiring a washout period. This approach avoids even the slightest discrepancies in the characteristics of the populations included in each trial arm.

Finally, although we made efforts to gather extensive information on patients, disease, and treatments at the molecular and clinical level, and established benchmarks for validation, it is important to acknowledge that our approach, like any modelling technique, was constrained by the available information at the time of the study (46, 165). While our models were deemed suitable based on the available data, the presented models and conclusions are subject to updates and improvements over time as new prospective data and information are generated. The SB and QSP models were built by incorporating the whole human protein network and a wide range of drug-pathology relationships (**Supplementary Table E** in the **S2 File**) (51). This approach achieved an accuracy exceeding 90%, demonstrating the models’ ability to generalize and accommodate new information beyond the specific data compiled for psoriasis and the studied drugs.

## 5 Conclusion

Our study provided patient-specific QSP models of the MoA of CZP, a PEGylated Fab humanized monoclonal antibody against TNF-alpha, in a vPop of patients with moderate-to-severe psoriasis, based on PBPK and SB models. The models reproduced clinical (i.e., PASI scores from different psoriasis treatments) and molecular (i.e., known psoriasis severity markers) efficacy features, supporting the use of these approaches to build hypotheses-generating models. The models’ analyses allowed to identify clusters of MoA solutions regardless of the dosing scheme, inferring the existence of dose-independent MoA differences between virtual patients, potentially involving developmental processes, such as angiogenesis and the Wnt pathway. The presented findings highlight the potential of *in silico* population- and patient-specific modeling approaches in advancing the study of diverse and complex diseases like psoriasis. These approaches enable detailed investigation of distinct pathophysiology and drug mechanisms. However, it is important to recognize that the results obtained from these models should be considered as hypotheses, requiring further prospective studies for clinical applicability. Nonetheless, such modeling approaches have the potential to reduce and refine pre-clinical and clinical experimentation and provide valuable data in the post-marketing setting.

## Data availability statement

The original contributions presented in the study are included in the article/**Supplementary Material**, further inquiries can be directed to the corresponding authors.

## Author contributions

Conceptualization: JM, BO, XD, CC, OS-M, PC-S, AM, DM-R, SA. Data curation: CS-V, GJ. Formal analysis: CS-V, GJ, VJ, JM. Funding acquisiton: JM. Investigation: CS-V, GJ. Methodology: CS-V, GJ, VJ, FG, JM, BO, XD. Project administration: CS-V, CC, OS-M. Resources: JM, CC, OS-M. Software: GJ, VJ, FG. Supervision: JM, BO, XD, CC, OS-M. Validation: JM, BO, XD, PC-S, AM, DM-R, SA. Visualization: CS-V, GJ. Writing – original draft preparation: CS-V, GJ. Writing – review & editing: VJ, FG, JM, BO, XD, CC, OS-M, PC-S, AM, DM-R, SA. All authors contributed to the article and approved the submitted version.

## Supplementary material

Supplementary Figures Data Sheet 1.PDF

Supplementary Tables Table 1.XLSX
