# Supplementary material for: A quantitative systems pharmacology model for certolizumab pegol treatment in moderate-to-severe psoriasis
Source: Front Immunol. 2023 Sep 20;14:1212981. doi: 10.3389/fimmu.2023.1212981 (PMC10552644; doi:10.3389/fimmu.2023.1212981)
Supplement: Supplementary Figures — Data Sheet 1.PDF [file DataSheet_1.pdf]

SUPPLEMENTARY FIGURES

Certolizumab pegol for moderate-to-severe psoriasis: A quantitative systems pharmacology model

AUTHORS: Pablo Coto Segura[1†], Cristina Segú-Vergés[2,3†], Antonio Martorell[4*], David Moreno-Ramírez[5], Guillem Jorba[2,3], Valentin Junet[2,6], Filippo Guerri[2,6], Xavier Daura[6,7,8], Baldomero Oliva[3], Carlos Cara[9], Olaya Suárez-Magdalena[9], Sonya Abraham[10], José Manuel Mas[2]

AFFILIATIONS:

[1]Hospital Vital Álvarez-Buylla, Mieres, Asturias, Spain

[2]Anaxomics Biotech SL, Barcelona 08007, Spain

[3] Structural Bioinformatics Group, Research Programme on Biomedical Informatics, Department of Medicine and Life Sciences, Universitat Pompeu Fabra, Barcelona 08003, Spain

[4] Hospital de Manises, Valencia, Spain

[5] Dermatology Department, University Hospital Virgen Macarena, Andalusian Health Service, University of Seville, Seville, Spain

[6]Institute of Biotechnology and Biomedicine, Universitat Autònoma de Barcelona, Cerdanyola del Vallès 08193, Spain

[7]Catalan Institution for Research and Advanced Studies (ICREA), 08010 Barcelona, Spain

[8]Centro de Investigación Biomédica en Red de Bioingeniería, Biomateriales y Nanomedicina, Instituto de Salud Carlos III, 08193 Cerdanyola del Vallès, Spain

[9]UCB Pharma, Madrid, Spain

[10]Imperial College, London, UK

[†]These authors equally contributed to this work.

* Corresponding author: antmarto@hotmail.com

Supplementary figures

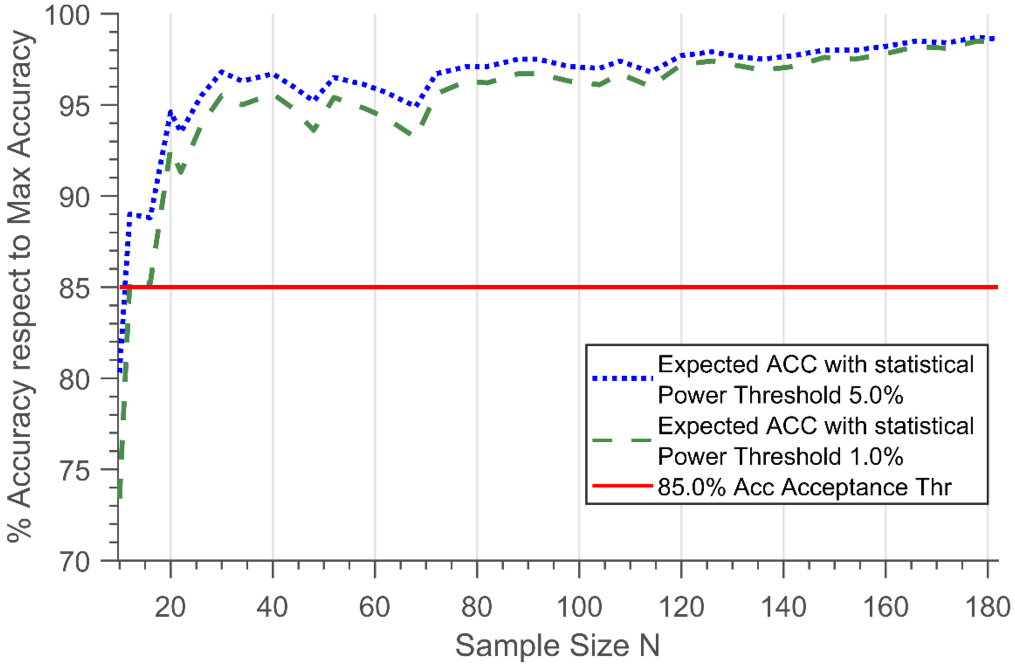

Supplementary Figure S1. Percentage of Max accuracy as a function of sample size. The dotted blue line corresponds to the mean % Max accuracy reached for each sample size subset at statistical power 95% and the discontinuous green line at 99%. The red line shows the 85% Max accuracy level.

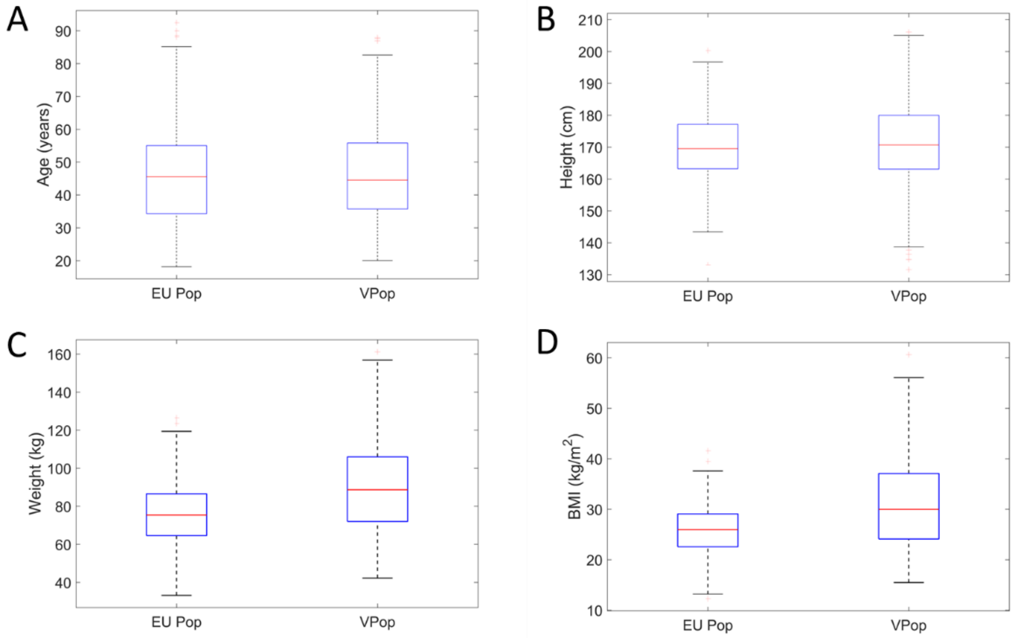

Supplementary Figure S2. Distribution representation of age, height, weight, and BMI demographic values between EU population and vPop.

BMI: Body mass index; EU: European; vPop: Virtual population

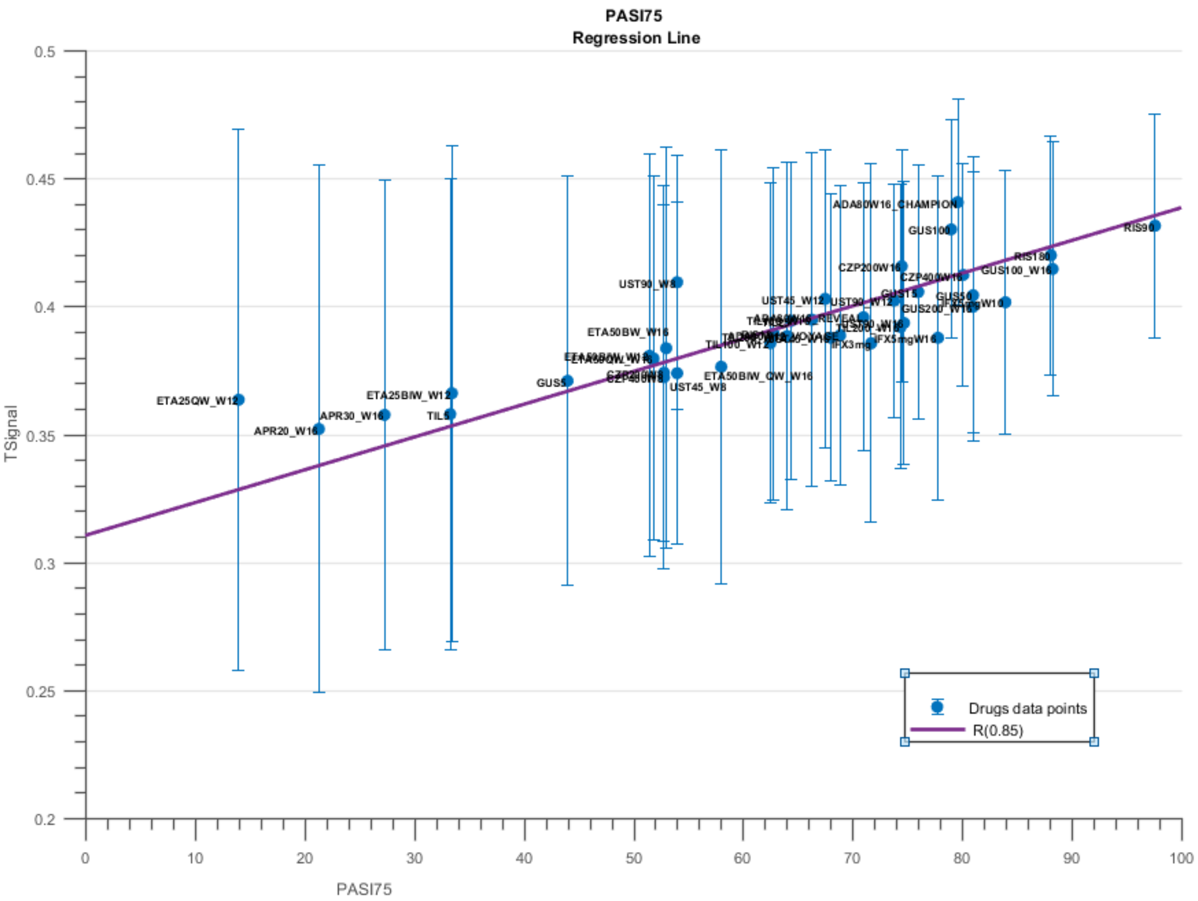

Supplementary Figure S3. Regression line between optimized psoriasis tSignal in relation to PASI75 values (R = 0.85).
